# How do our cells build their protein interactome?

**DOI:** 10.1038/s41467-018-05448-2

**Published:** 2018-07-27

**Authors:** Benoit Coulombe, Philippe Cloutier, Marie-Soleil Gauthier

**Affiliations:** 1 Translational Proteomics Research Unit, Montreal Clinical Research Institute, Montreal, H2W 1R7 QC Canada; 2 Department of Biochemistry and Molecular Medicine, Université de Montréal, Montreal, H3T 1J4 QC Canada

## Abstract

Chaperones are cellular factors that help in the folding of newly synthesized polypeptides (or clients) and, in some cases, ensure their integration within larger complexes. They often require non-client proteins, or co-chaperones, to help drive specificity to particular target polypeptides or facilitate the nucleotide hydrolysis cycle of some chaperones. The latest findings on the characterization of the PAQosome (Particle for Arrangement of Quaternary structure; formerly known as *R2TP/PFDL* complex) published recently in *Nature Communications* help to explain how this particular co-chaperone plays a central role in organizing our proteome into protein complexes and networks. The exploitation by the cell of alternative PAQosomes formed through the differential integration of homologous subunits, in conjunction with the use of several adaptors (specificity factors), provide the conceptual basis for interaction of multiple clients in a structure that is favorable to their simultaneous binding en route to protein complex and network assembly/maturation.

## Introduction

Modern biological research has revealed that the highly complex set of proteins that make up an organism—its proteome—is organized into multisubunit complexes and networks that regulate essential cell and tissue functions. This process of protein complexes assembly and wiring of interactome networks is still poorly understood.

In 2005, a proteomic screen for HSP90 interactors in yeast performed by Houry and colleagues^1^ identified a protein complex, named R2TP (Ruvb1, Ruvb2, Tah1, Pih1), which assists the molecular chaperone HSP90 in assembly of specific ribonucleoproteins (small nucleolar ribonucleoprotein)^2^. What turned out to be the mammalian equivalent of R2TP was later discovered as a complex that tightly associates with human RNA polymerase II to regulate its assembly^3–7^. In addition to an R2TP module similar to that found in *Sachharomyces cerevisiae*, the mammalian complex contains a group of additional factors including a Prefoldin-like module composed of classical prefoldins, prefoldin-like proteins, and some additional factors. The role of RUVBL1/2 components and the prefoldin-like module remain unclear. First called R2TP/Prefoldin-like (R2TP/PFDL), this complex was recently renamed PAQosome (Particle for Arrangement of Quaternary structure), to better reflect its function in HSP90-driven assembly/maturation of a number of key protein complexes and networks^6^. The exact mechanism by which the PAQosome assembles its client protein complexes is still unclear. The available data suggest that specific clients are recruited to the PAQosome, some directly and some indirectly via adaptors, and can then be assembled and stabilized by the platform formed by the PAQosome together with HSP90. As our cells must assemble thousands of protein complexes in a dynamically regulated manner, it is highly improbable that the original PAQosome is the only cellular particle responsible for assembly of all these complexes; alternative PAQosomes must exist.

**Fig. 1:**
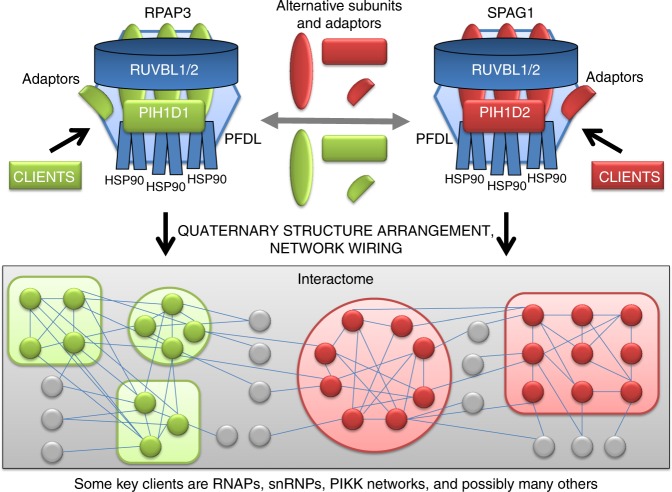
Representation of putative alternative PAQosomes. The PAQosome (originally R2TP/PFDL complex) exists in different flavors generated through the differential integration of homologous subunits (e.g., RPAP3 vs. SPAG1, PIH1D1 vs. PIH1D2, etc). The results of Martino et al.^9^ suggest that up to three RPAP3 may bind the RUVBL1-RUVBL2 scaffold, although this has not been confirmed in vivo. The existence of alternative PAQosomes along with the use of various specificity factors (adaptors) can theoretically multiply by many fold the number of clients they can act upon during quaternary structure arrangement, playing a central role in building up the interactome (protein complexes and networks). Whether alternative subunits modify the overall organization and composition of the PAQosome remains to be addressed experimentally

In a recent study, Bertrand and colleagues^8^ made what can be seen as a major breakthrough in this area. These authors discovered that some PAQosome subunits have homologous proteins encoded by the human genome that are able to form alternative PAQosome complexes with their own specific sets of clients (Fig. 1). Moreover, the data support the notion that alternative PAQosomes can harbor tissue specificity. Indeed, Maurizy et al.^8^ identified and characterized in some details a version of the PAQosome, enriched in testis, in which RPAP3 is likely replaced by the homologous protein SPAG1, and PIH1D1 by PIH1D2. These modest differences drastically alter the specificity of the resulting particle, which is now able to recognize a totally different set of clients such as liprin complexes. This specific PAQosome variant that they named R2SP was characterized in great details in their study. Nonetheless, their work on other PIH1D1 and RPAP3 isoforms and homologous proteins hints at the existence of the PAQosome in many other flavors, each assembling a number of specific complexes and networks. Moreover, the fact that the PAQosome associates in some cases with adaptor proteins to acquire client specificity while favoring diversity, tremendously increases the complexity and possibilities of protein clients. With these novel findings in hand, it becomes easier to imagine that the PAQosome is responsible for assembly of a vast number of protein complexes and networks in mammals. Although this remains to be supported by relevant data in the future, Maurizy et al.^8^ provides evidence that a modular, cell-specific PAQosome toolbox could be employed by cells.

Interestingly, the structural data in both Maurizy et al.^8^ and Martino et al.^9^ provides support to a model in which the organization of RPAP3 and PIH1D1 around the RUVBL hexamer can support recruitment of HSP90 and clients to the PAQosome (see Martino et al.^9^, Figure 8; and Fig. 1 herein), with sufficient versatility to explain client complexity.

Additional studies will be required to further characterize the PAQosome, the regulation of its subunit composition and mechanism of action, as well as the role of the PFDL module on PAQosome function.

## References

[1] Zhao R (2005). Navigating the chaperone network: an integrative map of physical and genetic interactions mediated by the Hsp90 chaperone. Cell.

[2] Boulon S (2008). The Hsp90 chaperone controls the biogenesis of L7Ae RNPs through conserved machinery. J. Cell Biol..

[3] Jeronimo C (2007). Systematic analysis of the protein interaction network for the human transcription machinery reveals the identity of the 7SK capping enzyme. Mol. Cell.

[4] Cloutier P (2009). High-resolution mapping of the protein interaction network for the human transcription machinery and affinity purification of RNA polymerase II-associated complexes. Methods.

[5] Boulon S (2010). HSP90 and its R2TP/Prefoldin-like cochaperone are involved in the cytoplasmic assembly of RNA polymerase II. Mol. Cell.

[6] Houry WA, Bertrand E, Coulombe B (2018). The PAQosome, an R2TP-based chaperone for quaternary structure formation. Trends Biochem. Sci..

[7] Cloutier P (2017). R2TP/Prefoldin-like component RUVBL1/RUVBL2 directly interacts with ZNHIT2 to regulate assembly of U5 small nuclear ribonucleoprotein. Nat. Commun..

[8] Maurizy C (2018). The RPAP3-Cterminal domain identifies R2TP-like quaternary chaperones. Nat. Commun..

[9] Martino F (2018). RPAP3 provides a flexible scaffold for coupling HSP90 to the human R2TP co-chaperone complex. Nat. Commun..

